# Giant Cell Tumour of Knee Popliteus Muscle Synovial Sheath

**DOI:** 10.7759/cureus.1996

**Published:** 2017-12-28

**Authors:** Cathal Mac Dhaibheid, MN Baig, Desmond Harrington, Fintan J Shannon

**Affiliations:** 1 Trauma & Orthopaedics, Galway University Hospital

**Keywords:** giant cell tumour of the tendon sheath, pigmented villonodular tumour of the tendon sheath

## Abstract

The giant cell tumour of the tendon sheath (GCTTS) is the second most common soft tissue benign tumour and rarely presents in the knee. We report a rare presentation of a GCTTS in the knee with corresponding magnetic resonance imaging (MRI), an arthroscopic picture, and histological presentation. It is a rare occurrence but should be considered as a differential in atraumatic knee pain presentation. This case report gives a classic picture of its presentation, diagnosis, and histopathology.

## Introduction

Giant cell tumour of tendon sheath (GCTTS), also known as a pigmented villonodular tumour of the tendon sheath (PVNS), is a benign tumour composed of synovial-like mononuclear cells of the joint, the tendon sheaths, and the mucous bursae [1]. It usually presents in the third to fifth decade of life and is the second most common soft tissue benign tumour following ganglion cyst tumours [2].

GCTTS is most frequently located paratendinous in the sheath of the flexor tendons of the hand. The second most common location is in the joints such as the hip, ankle, and shoulder [2]. GCTTS is rarely located in bursae. Clinically, it can present as pain, swelling, effusion, enlarging mass or it may be asymptomatic. But like many other conditions, it can present with atypical sign and symptoms [3]. Ultrasonography and magnetic resonance imaging (MRI) is the most characteristic diagnostic tools. X-rays are useful in patients with bony erosions, which is approximately 5% of GCTTS patients [4].

## Case presentation

A 34-year-old woman presented to the orthopaedic elective clinic with concerns of global pain in the left knee. She had no history of fall, trauma or injury. The pain and its resultant disability prevented her from working, and the pain gradually intensified. There was no history of giving away but rare episodic locking was present.

On examination, we noted a small swelling on the posterolateral aspect of the knee. Her range of motion for the knee was 0 to 110 degrees which is decreased. She was moderately tender over the lateral aspect of her left knee.

**Figure 1:**
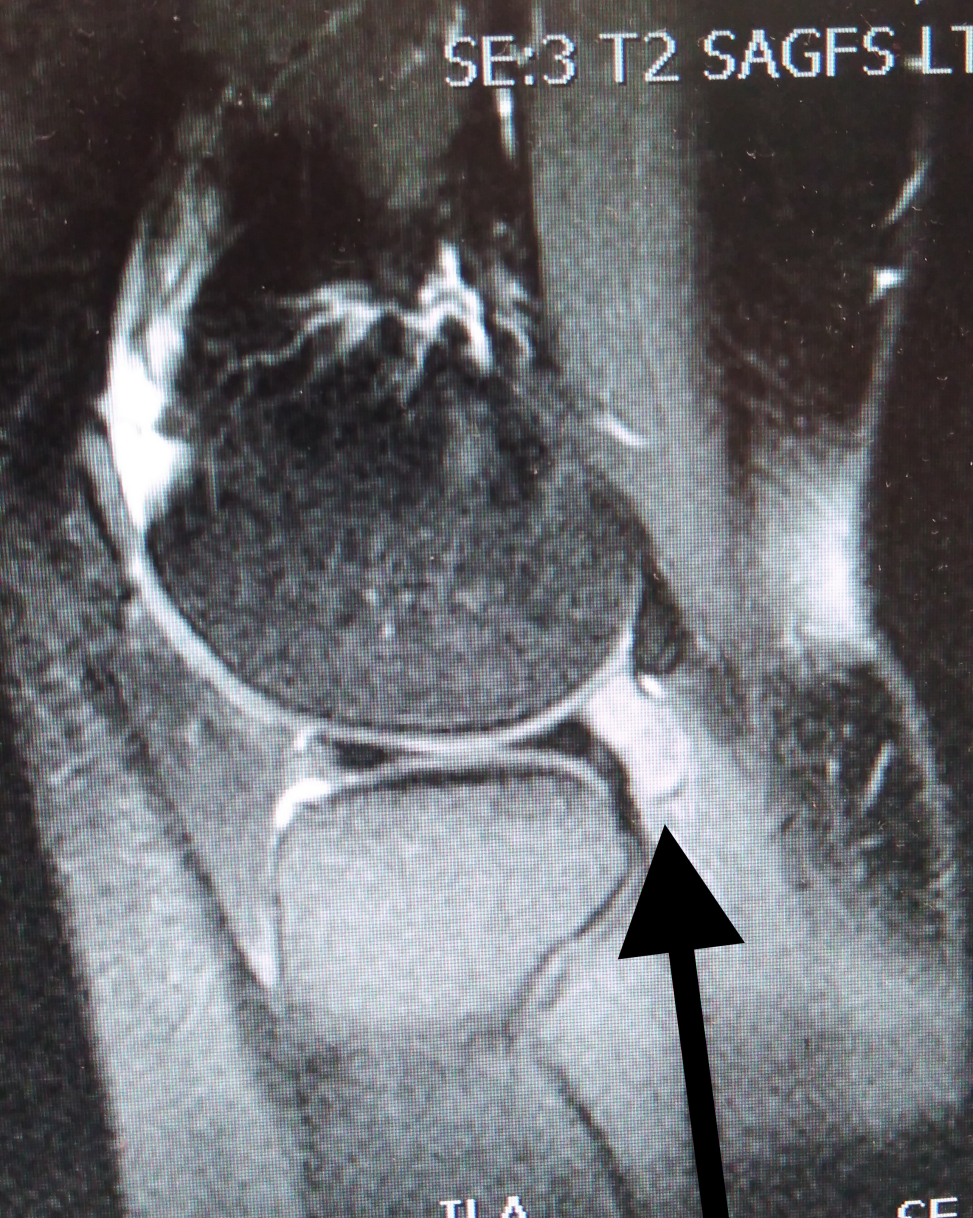
MRI of giant cell tumour MRI (T2 weighted) of giant cell tumour of popliteus tendon sheath. MRI: Magnetic resonance imaging.

**Figure 2:**
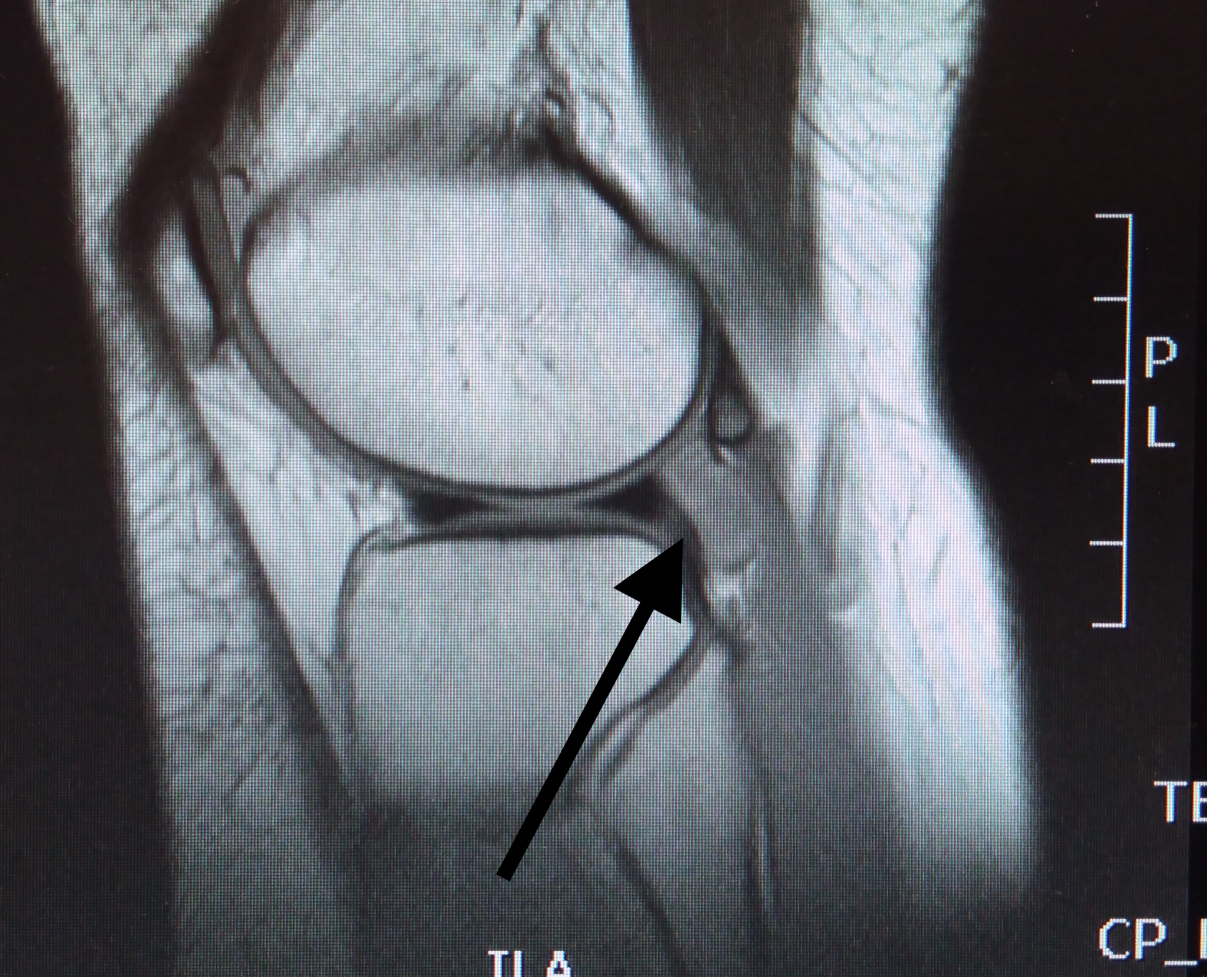
MRI of giant cell tumour MRI (T1 weighted) sequence: 2.6 cm in craniocaudal length, 2 cm in the transverse plane. MRI: Magnetic resonance imaging.

An MRI revealed a soft tissue mass posterior to the lateral meniscus, adjacent to the popliteus tendon measuring 2.6 cm in craniocaudal length, 2 cm in the transverse plane, and 0.9 cm in the anteroposterior plane (Figures 1, 2).

**Figure 3:**
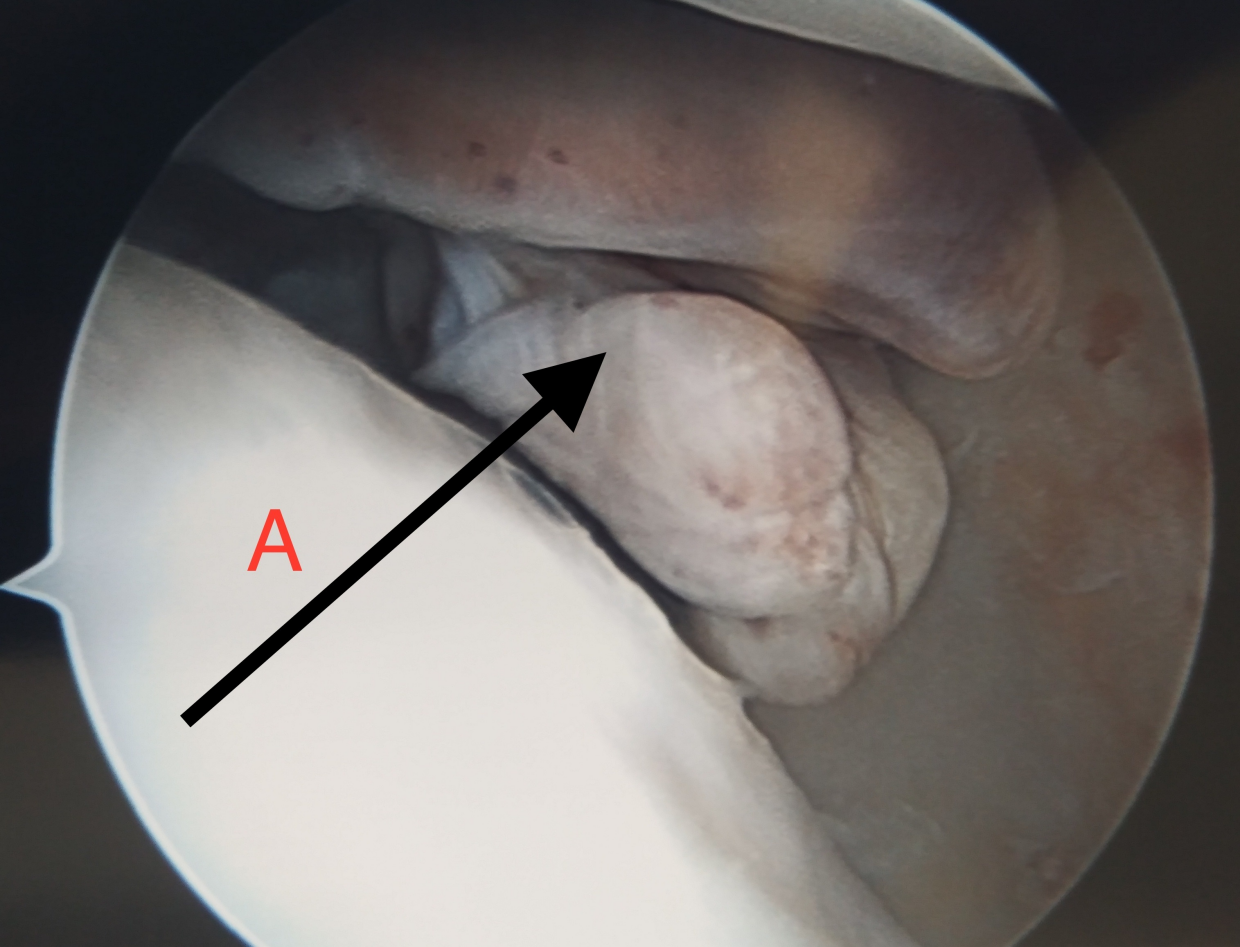
Arthroscopic picture A - Arthroscopic picture showing the giant cell tumour tendon sheath (GCTTS) lesion.

We scheduled her for a knee arthroscopy, during which we noted a mass in the posterolateral aspect of knee joint posterior to the lateral meniscus. We debulked the tumour and sent it for biopsy. There was no other abnormality seen during arthroscopy (Figure 3 ).

**Figure 4:**
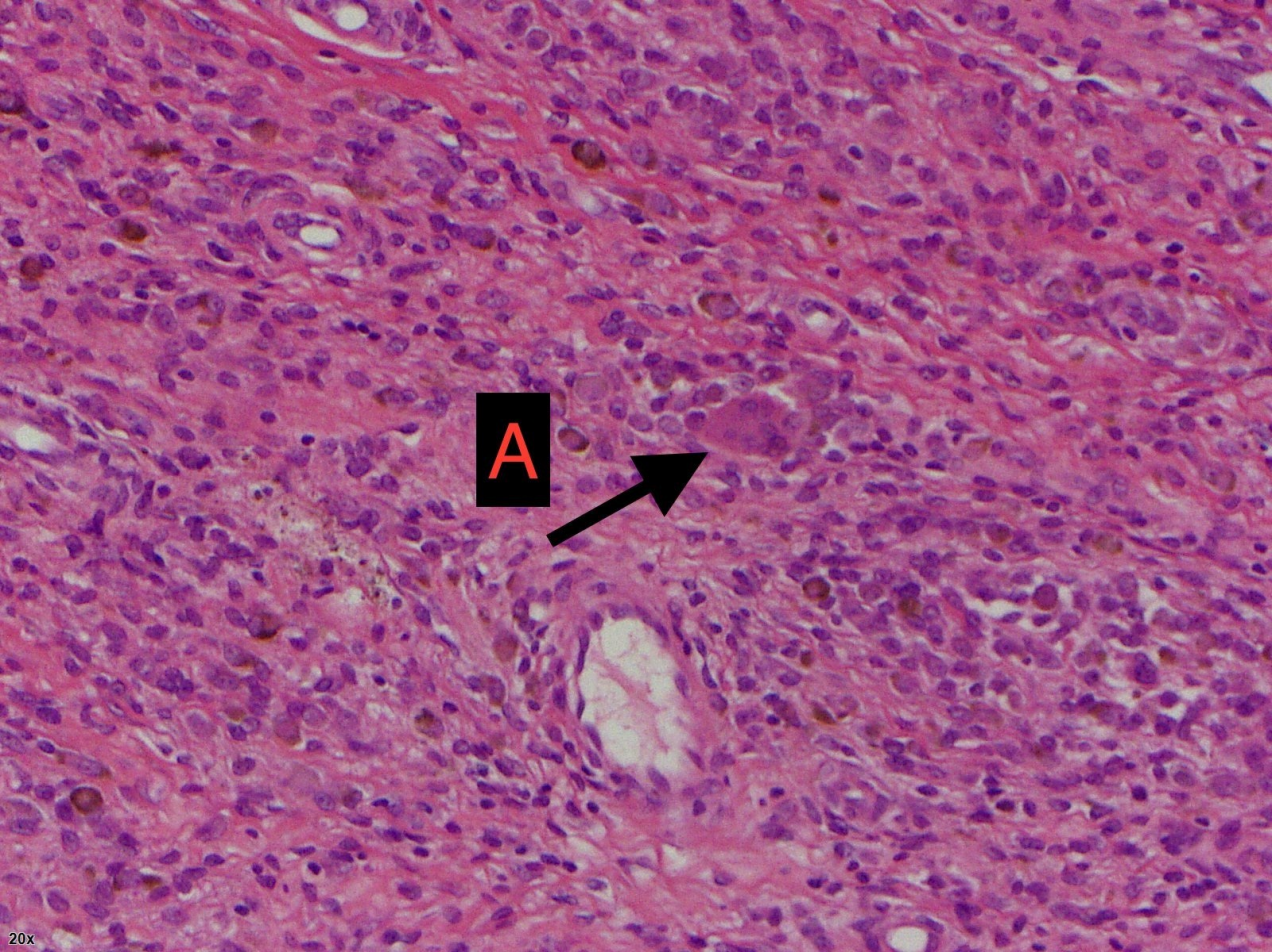
Histology slide A - Multi-nucleated giant cells in mononuclear background.

**Figure 5:**
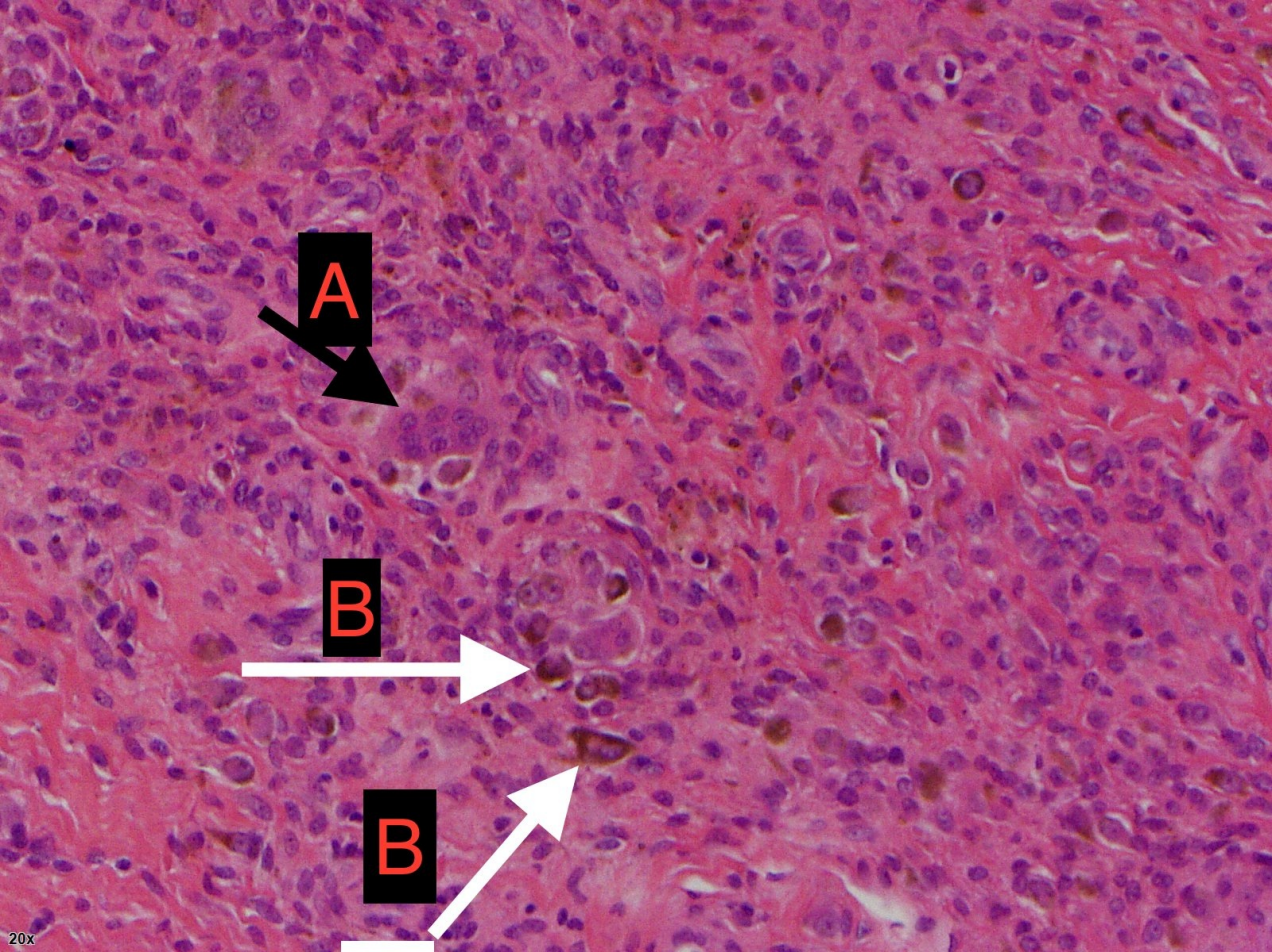
Histology slide A - Giant cells. B - Hemosiderin-laden macrophages.

The histology report revealed a lobulated piece of highly cellular tissue composed of a polymorphous cell population including large epithelioid cells (Figures 4, 5 ). We also noted xanthoma cells and hemosiderin-laden macrophages present (Figure 5). The cells are CD68/CD163 positive and CD34/desmin negative. The overall features were consistent with GCTTS.

The patient presented significant clinical improvement at the two-week follow-up evaluation. She is undergoing rehabilitation and we are following her in our clinic routinely.

## Discussion

GCTTS was first described by Chassaignac in 1852 as fibrous xanthoma, but the names have changed over time [5]. There is no consensus on its aetiology; the literature reports both inflammatory origins and neoplastic origins. The most common location is the fingers (the index is most common, followed by the middle finger). The most common symptom is localised tenderness followed by bony erosion and numbness. The recurrence rate of GCTTS ranges from 4% to 44%.

This case is interesting as tenosynovial tumours are a rare occurrence, especially in large weight-bearing joints, like knee [6]. The literature advocates both open and arthroscopic resection with or without synovectomy. We started our procedure arthroscopically with an open mind that if required we may need to convert it to open resection. But we were able to resect the whole tumour arthroscopically. In addition to standard knee arthroscopy incisions (Anteromedial, anterolateral) we introduced a posterolateral portal as well to get a better access to the lesion. The absence of gene nm23 has been associated with high recurrence rate, but it is not conclusive [7].

## Conclusions

While GCTTS is not a very rare condition itself, its presentation in the knee is rare and should be kept in mind when considering differential diagnoses in patients with signs and symptoms of meniscal injury with atraumatic history. MRI is the investigation of choice and arthroscopic resection is the treatment of choice. Clinical features, radiological assessment, and histopathological confirmation are necessary for the final diagnosis.
